# Serum Calprotectin a Potential Biomarker in Juvenile Idiopathic Arthritis: A Meta-Analysis

**DOI:** 10.3390/jcm10214861

**Published:** 2021-10-22

**Authors:** Emma Altobelli, Paolo Matteo Angeletti, Reimondo Petrocelli, Giuseppe Lapergola, Giovanni Farello, Giovanni Cannataro, Luciana Breda

**Affiliations:** 1 Department of Life, Public Health and Environmental Sciences, University of L’Aquila, 67100 L’Aquila, Italy; paolomatteoangeletti@gmail.com (P.M.A.); giovanni.farello@univaq.it (G.F.); 2 Rianimazione e TIPO Cardiochirurgica, Ospedale G. Mazzini, Local Health Unit, 64100 Teramo, Italy; 3 ASREM, 86100 Campobasso, Italy; reimondo.petrocelli@asrem.it; 4 Department of Pediatrics, Neonatal Intensive Care Unit, G. D’Annunzio University, 66100 Chieti, Italy; g.lapergola@gmail.com; 5 Department of Pediatrics, University of L’Aquila, 67100 L’Aquila, Italy; 6 Department of Neuroscience and Imaging, Section of Diagnostic Imaging and Therapy- Radiology Division, G. D’Annunzio University, 66100 Chieti, Italy; cannatarogiovanni@gmail.com; 7 Department of Pediatrics, Pediatric Rheumatology Unit, G. D’Annunzio University, 66100 Chieti, Italy; luciana.bredach@gmail.com

**Keywords:** serum calprotectin, systematic review, meta-analysis, juvenile idiopathic arthritis, biomarkers

## Abstract

Juvenile idiopathic arthritis (JIA) is the most common inflammatory chronic disease affecting children and adolescents. Today, there are no specific biomarkers of inflammation. Therefore, it is important to identify new markers as predictors of disease activity. Recently, some researchers have directed their interest toward a protein, calprotectin (CLP), as a potential biomarker. The primary objective of our systematic review and meta-analysis was to analyze the possible role of CLP in JIA. Method: A literature search was conducted using PubMed, EMBASE, Scopus, Science Direct on 10 August 2021. The selection of studies was made using the PRISMA 2020 guidelines. Cohen’s d with 95% CI and *p*-value were used as a measure of effect size. The random effects model was used to account for different sources of variation among studies. Heterogeneity was assessed using Q statistics and I^2^. The publication bias was analyzed and represented by a funnel plot, and funnel plot symmetry was assessed with Egger’s test. Results: Our results at follow-up showed a statistically significant difference between patients with active disease compared to patients with inactive disease: 0.39 (0.16; 0.62), *p* = 0.001; without statistical heterogeneity. Another important aspect that emerged were the differences between the systemic disease form and any form of inactive disease showing a different concentration of calprotectin: 0.74 (0.40; 1.08), *p* < 0.001; without statistical heterogeneity. On the other hand, meta-regression analyses performed on gender, age, duration of disease, percentage of patients with ANA+ or RF+, medium value of ESR or CRP were not statistically significant. A statistically significant difference in serum calprotectin concentration between patients with JIA and healthy controls were observed. In fact, it presented lower values in the control group. Conclusions: The use of serum CLP could represent, in the future, a useful tool in JIA in order to stratify disease activity more accurately and may aid a more tailored approach to drug of choice in children with JIA. Further studies are needed to evaluate CLP as a predictor of flare in combination with other potential biomarkers of subclinical disease activity.

## 1. Introduction

Juvenile idiopathic arthritis (JIA) is the most common autoimmune/inflammatory chronic disease affecting children and adolescents. JIA comprises a heterogeneous group of joint diseases with distinct clinical phenotypes, disease courses, and outcomes. It is known that JIA can be associated with morbidity and mortality and represents a cause of short-term and long-term disability. The epidemiology is variable worldwide with incidence rates ranging from 1.6 to 23/100.000 [1,2]. In the past two decades, there have been important therapeutic advances in the management of JIA, however, to date, we are not yet able to predict the course of the disease, the therapeutic response, and relapses after drug discontinuation. Therefore, there is a great need for biomarkers to guide clinical decisions such as starting, switching, or tapering methotrexate or biologic DMARDs [3].

In general, the inflammatory microenvironment of chronic arthropathies is quite varied and current knowledge of the relationships and interplay between markers is limited [4].

Recently, some researchers have directed their interest toward a protein, calprotectin (CLP), as a potential biomarker for disease activity [5,6,7,8,9,10,11,12,13]. 

CLP is a granulocyte and monocyte complex of calcium- and zinc-binding proteins that belong to the S100 protein family and is released during cell activation and turnover [14,15,16]. 

It is also referred to as S100A8/A9, MRP8/MRP14 (myeloid-related-protein 8/14), calgranulin A/B, L1 protein, and cystic fibrosis antigen and is composed by two subunits of 8 kDa and 14 kDa, respectively.

Both MRP8 and MRP14 form a stable non-covalent associated heterodimer, which is essential for the tetramerization in CLP (MRP8/MRP14) [14]. 

Interestingly, MRP8/MRP14 proteins originate from inflammatory cells (activated phagocytes) within the inflamed synovium and thus correlate strongly with local disease processes in joints, even when no systemic involvement occurs [17]. 

The MRP8/MP14 serum levels correlate with disease activity in JIA patients [17] and could be used to identify subclinical disease activity [18,19,20,21]. 

Moreover, musculoskeletal ultrasound (MSUS) is used increasingly as a useful imaging tool for evaluating JIA patient disease activity [22,23]. Many studies have therefore demonstrated that clinical examination in JIA may underestimate the extension of synovitis and sometimes cannot be accurate, whereas MSUS could improve the sensitivity and the accuracy in the detection of the exact sites of inflammation in the joint [24,25]. Implementation of clinical examination with MSUS in children with JIA could lead to important implications in therapeutic decisions (i.e., indication to a second line drug or biologic treatment, or exact location of intra-articular corticosteroid injections) and monitoring of treatment efficacy [22,23]. It has been recently argued that remission of JIA defined on clinical grounds does not couple with remission defined with imaging [26,27]. However, the clinical significance and prognostic value of this finding is unclear, as the presence of MSUS abnormalities including PD signal in patients with clinically defined inactive disease did not predict subsequent synovitis flare [28]. 

The present study provides (i) a systematic review and a meta-analysis of the possible role of CLP serum protein in JIA, and (ii) MSUS as a useful imaging tool for evaluating a JIA patient’s disease activity.

## 2. Material and Methods

**Figure 1:**
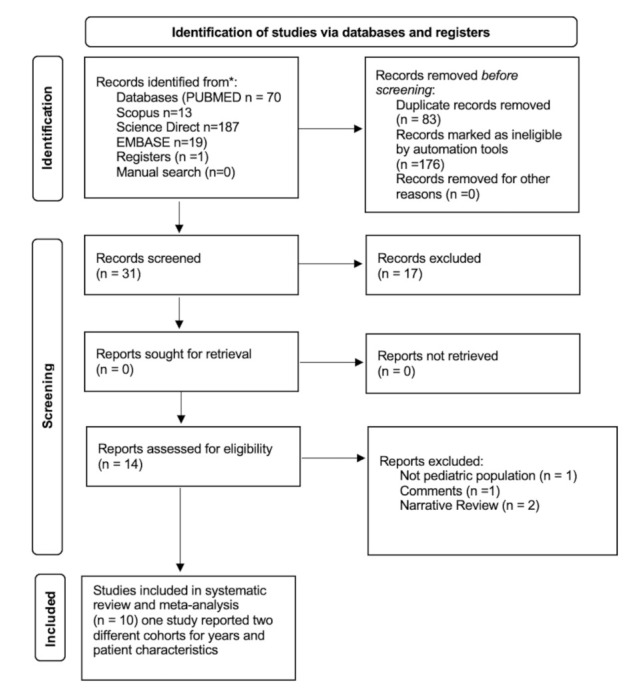
PRISMA flowchart.

A literature search was conducted on PubMed, EMBASE, Scopus, Science Direct on 10 August 2021 with the following keywords: “arthritis, juvenile”[MeSH Terms] OR (“arthritis, juvenile”[MeSH Terms] OR (“arthritis”[All Fields] AND “juvenile”[All Fields]) OR “juvenile arthritis”[All Fields] OR (“juvenile”[All Fields] AND “arthritis”[All Fields]))) AND (“s100a8 a9”[All Fields] OR MRP8/14 OR (“calprotectine”[All Fields] OR “leukocyte l1 antigen complex”[MeSH Terms] OR (“leukocyte”[All Fields] AND “l1”[All Fields] AND “antigen”[All Fields] AND “complex”[All Fields]) OR “leukocyte l1 antigen complex”[All Fields] OR “calprotectin”[All Fields]) OR “leukocyte l1 antigen complex”[MeSH Terms]) (“serum”[MeSH Terms] OR “serum”[All Fields] OR “serums”[All Fields] OR “serum s”[All Fields] OR “serumal”[All Fields]) AND (“calprotectine”[All Fields] OR “leukocyte l1 antigen complex”[MeSH Terms] OR (“leukocyte”[All Fields] AND “l1”[All Fields] AND “antigen”[All Fields] AND “complex”[All Fields]) OR “leukocyte l1 antigen complex”[All Fields] OR “calprotectin”[All Fields]) AND (“arthritis, juvenile”[MeSH Terms] OR (“arthritis”[All Fields] AND “juvenile”[All Fields]) OR “juvenile arthritis”[All Fields] OR (“juvenile”[All Fields] AND “idiopathic”[All Fields] AND “arthritis”[All Fields]) OR “juvenile idiopathic arthritis”[All Fields]) AND (“diagnostic imaging”[MeSH Subheading] OR (“diagnostic”[All Fields] AND “imaging”[All Fields]) OR “diagnostic imaging”[All Fields] OR “ultrasound”[All Fields] OR “ultrasonography”[MeSH Terms] OR “ultrasonography”[All Fields] OR “ultrasonics”[MeSH Terms] OR “ultrasonics”[All Fields] OR “ultrasounds”[All Fields] OR “ultrasound s”[All Fields]). A manual search was also conducted. Only studies published in English over the previous 10 years were considered. The papers were selected by two independent reviewers (P.M.A. and G.L); a methodologist (E.A.) resolved any disagreements. The selection of studies was made using the PRISMA 2020 guidelines [29] (Figure 1). Quality control of the systematic review was performed using the PRISMA checklist [29] (Supplementary Table S1). Supplementary Table S2 summarizes the data of primary studies stratified by study design, diagnostic test used, and baseline values of serum calprotectin according to patients (active or inactive) and data about therapy. 

**Figure 2:**
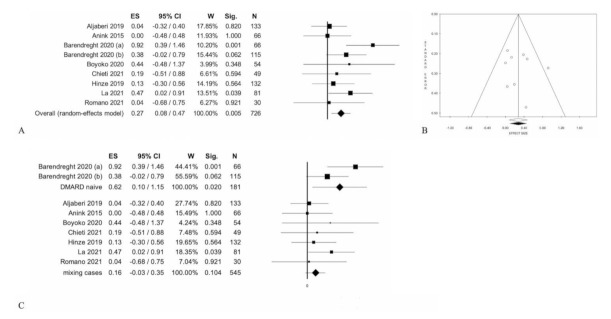
Active disease (pauci and polyarticular forms) vs. inactive disease: (**A**) Forest Plot of overall cases; (**B**) Funnel Plot; (**C**)forest plot according baseline therapy.

**Figure 3:**
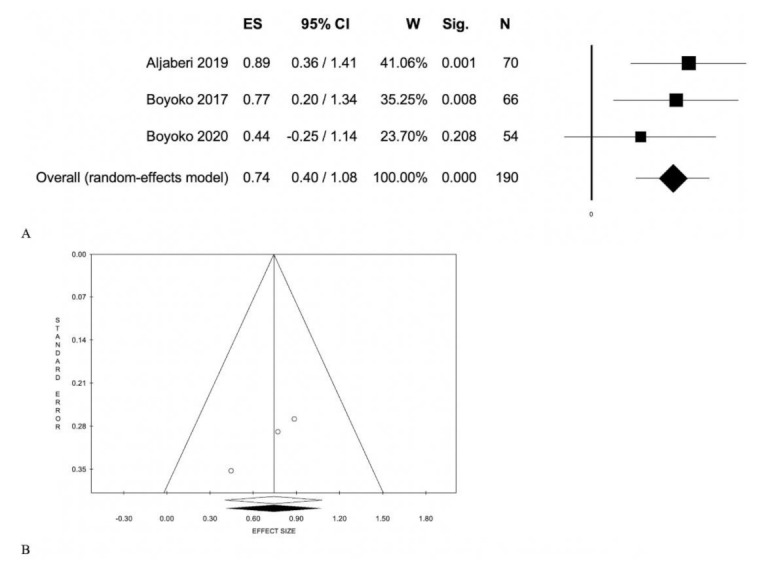
Active systemic disease vs. any inactive disease form (systemic and articular): (**A**) Forest plot; (**B**) Funnel plot.

**Figure 4:**
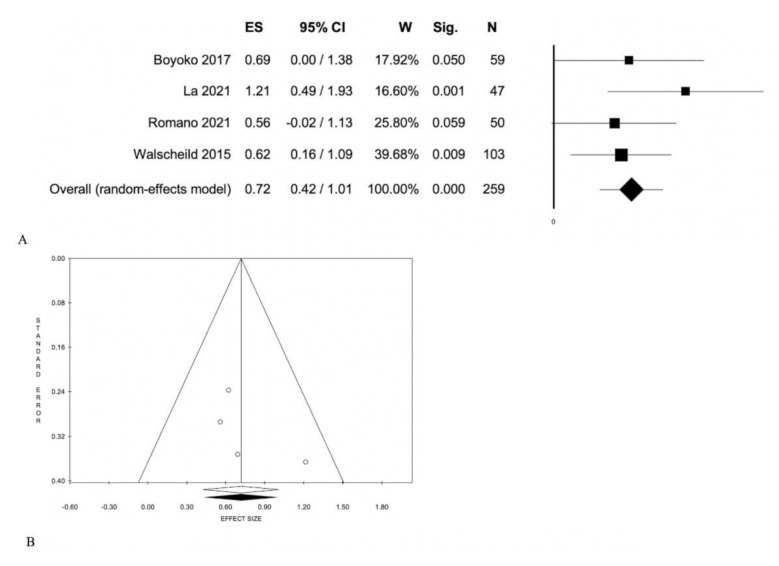
Subjects with any form (active/non active; systemic/non systemic) vs. healthy controls: (**A**) Forest Plot; (**B**) Funnel Plot.

**Figure 5:**
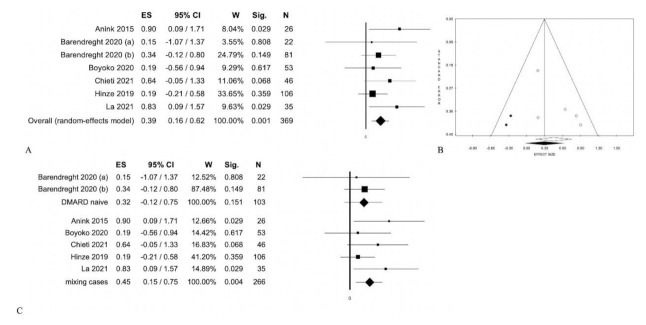
Calprotectin levels at 3–6 months (follow-up) between active and inactive disease: (**A**) Forest Plot of overall cases; (**B**) Funnel Plot; (**C**)forest plot according baseline therapy.

For case-control and cohort studies, the bias analysis was performed using the Newcastle–Ottawa Scale [30] (Supplementary Tables S3 and S4). Results of meta-analyses are shown in Table 1 and Figure 2, Figure 3, Figure 4 and Figure 5 and S1. Table 2 shows the results of the meta-regressions. 

### Statistical Analysis

Data from the Chieti Registry were analyzed using the following statistical models: normality distribution was assessed with the Shapiro–Wilk test and the Wilcoxon sign-rank test was used for the matched data. All data are presented in Supplementary Table S1. The echography score was evaluated according to Magni-Manzoni [22]. For meta-analysis, Cohen’s d with 95% CI and *p*-value were used as the measures of effect size. Data from primary studies presented as medians and interquartile ranges were transformed into mean and standard deviation (SD), as described by Pudar Hozo et al. [31].

The random effects model was used to account for different sources of variation among studies. Heterogeneity was assessed using Q statistics and I^2^. The publication bias was analyzed and represented by a funnel plot, and funnel plot symmetry was assessed with Egger’s test [32]. Publication bias was checked using the trim and fill procedure and PROMETA 3 software (IDo Statistics-Internovi, Cesena, Italy) was used. Meta-regression analyses were utilized for the following variables: % of males, age, duration of disease, ANA positivity, rheumatoid factor positivity, values of C-reactive protein (CRP), and erythrocyte sedimentation rate (ESR). 

We identified 290 references. Of these, 176 were removed by automation tools (filters applied: English language, 10 years and age <19 years) and 83 as duplicates. The remaining 31 abstracts were examined. Of these, 17 were eliminated. Finally, 14 reports were evaluated for eligibility criteria, four of which were excluded for the following reasons listed in Figure 1. 

Eight of the 10 studies compared active versus inactive disease (pauci-articular and polyarticular forms) [6,8,9,10,11,12,13]. In particular, one study considered two different groups of patients with different duration of disease, and they enrolled in two different times [9]. Their comparison at baseline shows that there are serum calprotectin differences between patients with active and inactive disease: 0.27 (0.08;0.47), *p* < 0.005; without statistical heterogeneity (Table 1, Figure 2). However, when selecting patients based on therapy status, there are no statistically significant differences between active and inactive patients: 0.16 (−0.03: 0.40), *p* = 0.104; without statistical heterogeneity (Table 1, Figure 2) [8,9,10,11,12,13]. Meta-regression analyses performed on gender, age, duration of disease, percentage of patients with ANA+ or RF+, medium value of ESR or CRP were not statistically significant (Table 2).

Of the 10 studies analyzed, three performed [7,8,12] differences between systemic disease form and any form of inactive disease showing a different concentration of calprotectin: 0.74 (0.40; 1.08), *p* < 0.001; without statistical heterogeneity (Table 1, Figure 3).

Of all studies included in the meta-analysis, four reported the comparison of serum calprotectin levels among patients suffering from any form of JIA versus healthy controls [5,6,7,8,11] (Table 1, Figure 4). Although two studies compared adult subjects [5,8], the results showed a statistically significant difference: 0.72 (0.42; 1.01), *p* < 0.001; without statistical heterogeneity (Table 1, Figure 4). Additionally, in this case, the meta-regressions did not show statistical significance (Table 2).

Six studies that considered calprotectin levels at follow-up were evaluated in separate meta-analysis. Among these, Barendreght et al. [9] enrolled naive patients stratified into two groups (Table S1). Our results at follow-up showed a statistically significant difference between patients with active disease compared to patients with inactive disease: 0.39 (0.16; 0.62), *p* = 0.001; without statistical heterogeneity (Table 1, Figure 5) [9,10,11,12,13]. Later, we stratified the six studies with respect to therapy at baseline. In Barendreght’s study, we did not observe a statistically significant difference in naive patients: 0.32 (−0.12; 0.75), (*p* = 0.151) (Table 1, Figure 5) [9]. On the other hand, patients with different therapies at baseline showed a statistically significant difference: 0.45 (0.15;0.75), *p* = 0.004 (Table 1, Figure 5 [10,11,12,13]. Meta-regression analyses highlighted no statistically significant differences (Table 2).

Finally, only two studies reported the concordance between MSUS and calprotectin levels: Romano et al. and patients on the Chieti Registry [2,6] to evaluate the relation between serum calprotectin values with clinical criteria (JADAS or Wallace) and ultrasound criteria. In all of these analyses, there were no differences between active and inactive disease, respectively 0.15 (−0.29; 0.58, *p* = 0.509) and 0.26 (−0.17; 0.70, *p* = 0.237) (Supplementary Figure S1).

## 3. Discussion

One the current unsolved problem in pediatric rheumatology is identifying predictive markers of disease activity, therapeutic response, and relapse upon discontinuation of treatment. To our knowledge, this is the first systematic review with a meta-analysis evaluating the role of serum CLP as a possible predictor marker in the management of JIA. Studies included in our meta-analysis point out the potential usefulness of this protein as a prospective biomarker for disease outcome. This hypothesis is based on previous researchers that have demonstrated a high concentration of S1000A8/S100A9 in synovial fluid obtained from an inflamed joint [17,33,34,35] that significantly decreased after intra-articular triamcinolone therapy in JIA [17,18].

The most important and interesting aspects in our meta-analysis were: first, serum calprotectin appears to be indisputably higher in active systemic-onset juvenile arthritis (SoJIA) (Figure 3). Our results showed a different concentration of calprotectin between SoJIA and any form of inactive disease: 0.74 (0.40; 1.08), *p* < 0.001. 

SoJIA is a particular subtype of JIA, different from other subtypes for its extra-articular features such as spiking fever, evanescent rash, and serositis with elevated parameters of inflammation. Upon presentation, SoJIA may be difficult to differentiate from other causes of fever of unknown origin (FUO) such as severe infections, malignancy, and other autoimmune or inflammatory diseases. S100A8/9 serum high concentrations can be found neither in other forms of arthritis nor in other causes of FUO [36]. These observations suggest that CLP is not only a marker of disease activity in SoJIA, but it can also be considered as a valid tool in differential diagnosis with other pathologies. This form is highly impactful from a clinical point of view, as evidenced by Lee et al. [37]. In this category of patients, the use of calprotectin could be extremely useful in monitoring anti-inflammatory and/or immunosuppressive therapy, minimizing complications. 

Second, there is a statistically significant difference in serum calprotectin concentration between patients with JIA and healthy controls, who presented lower values. Therefore, CLP is able to discriminate healthy subjects from those affected by JIA (Table 2, Figure 4). Third, our results clearly showed differences in serum calprotectin between patients with active versus inactive disease (*p* < 0.005).

On the other hand, it is important to underline that meta-regression analyses performed on gender, age, duration of disease, percentage of patients with ANA+ or RF+, medium value of ESR or CRP showed no statistically significant results.

Finally, it is important to highlight the results obtained from the analysis biomarker between the biomarker and joint ultrasound (Figure S1). In these studies, CLP with a JADAS score and MSUS were analyzed. 

These studies did not show any differences with respect to calprotectin values. We could attribute these results to two reasons: both ultrasound studies had patients with pauci-articular forms and undergoing therapy. In addition, they had a small sample size, insufficient to assert these findings with certainty. However, this approach appears to be a very promising technique for highlighting an early inflammatory state.

## 4. Conclusions

The use of serum CLP could represent, in the future, a useful tool in JIA in order to stratify disease activity more accurately and it may aid a more tailored approach to drug choice in children with JIA. However, a limitation present in the primary studies consist of not taking into account the stratification of the groups in reference to the presence or absence of therapy. 

Therefore, further studies are needed to evaluate CLP as a predictor of flare in combination with other potential biomarkers of subclinical disease activity.

Moreover, it would be useful to have the data available for each patient in order to be able to carry out an individual meta-analysis to improve the quality of the data.

## Figures and Tables

**Table 1:** Meta-analysis results.

Publication Bias				Heterogeneity					Publication Bias					
									Egger’s	Begg and Mazdumdar’s		Fail Safe		Rosenthal
Outcome	Effect Size	CI	*p* Value	Q	I2	*p* Value	T2	T	T	*p* Value	Z	*p* Value	No.	No.
**Active disease (pauci and polyarticular forms) vs. Inactive disease**														
*Overall cases (k = 9)* [6,8,9,10,11,12,13]	0.27	0.08; 0.47	0.005	10.5	23.78	0.232	0.02	0.14	0.47	0.654	0.83	0.404	15	55
*Mixing cases (k = 7)* [8,9,10,11,12,13]	0.16	−0.03; 0.40	0.104	3.17	0.00	0.787	0.00	0.00	0.54	0.609	1.05	0.293	0	45
*DMARDS naïve cases (k = 2)* [9]	0.62	0.10; 0.15	0.020	2.46	59.42	0.09	0.09	0.29	-	-	-	-	-	-
ANOVA Q *p* = 0.101														
**Active systemic disease vs. any inactive disease form (systemic and articular) (k = 3)** [7,8,12]	0.74	0.40; 1.08	<0.001	2.29	0.00	0.319	0.01	0.12	−46.28	0.008	−1.57	0.117	11	25
**Subjects with any form (active/non active; systemic/non systemic) vs. healthy controls (k = 4) ** [5,6,7,11]	0.72	0.42; 1.01	<0.001	2.30	0.00	0.513	0.00	0.00	1.24	0.304	1.36	0.174	21	30
**Calprotectin levels at 3–6 months (follow-up) between active and inactive disease**														
*Overall cases (k = 7)* [9,10,11,12,13]	0.39	0.16; 0.62	0.001	4.87	0.00	0.561	0.00	0.00	1.22	0.276	1.05	0.293	16	45
*Mixing cases (k = 5)* [10,11,12,13]	0.45	0.15; 0.75	0.001	4.62	13.44	0.328	0.06	0.23	2.06	0.132	1.47	0.142	10	35
*DMARDS naïve cases (k = 2)* [9]	0.32	−0.12; −0.75	0.151	0.08	0.00	0.777	0.00	0.00	-	-	-	-	-	-
ANOVA Q *p* = 0.681														

**Table 2:** Meta-regression results.

Variables	K	Intercept	Slope	*p* Value
Active disease (pauci and polyarticular forms) vs. Inactive disease				
Gender	6	−0.18	0.01	0.106
Age	4	−0.37	0.06	0.468
Duration of disease	5	0.19	0.01	0.953
ANA+	5	0.20	0.00	0.824
RF+	6	0.39	−0.02	0.148
ESR	5	−0.11	0.01	0.137
CRP	3	0.10	0.03	0.108
Subjects with any form (active/non active; systemic/non systemic) vs. healthy controls				
Gender	4	0.28	0.01	0.356
ANA+	3	0.87	0.00	0.776
ESR	3	0.36	0.02	0.397
CRP	3	0.49	0.06	0.213
Calprotectin levels at 3–6 months (follow-up) between active and inactive disease				
Gender	4	0.28	0.01	0.365
ANA+	3	0.87	0.00	0.766
ESR	3	0.36	0.02	0.397
CRP	3	0.49	0.06	0.213

## Supplementary Materials

The following are available online at https://www.mdpi.com/article/10.3390/jcm10214861/s1, Table S1: PRISMA Checklist. Table S2: Characteristics of included studies in systematic review and meta-analysis. Table S3: Risk of bias of case-control studies according to Newcastle–Ottawa. Table S4: Risk of bias of longitudinal studies according to Newcastle–Ottawa. Figure S1: Calprotectin levels according to clinical criteria and echography score.
