# Supplementary material for: Serum Calprotectin a Potential Biomarker in Juvenile Idiopathic Arthritis: A Meta-Analysis
Source: J Clin Med. 2021 Oct 22;10(21):4861. doi: 10.3390/jcm10214861 (PMC8584429; doi:10.3390/jcm10214861)
Supplement: Supplementary file 1 [file jcm-10-04861-s001.zip › jcm-1419468-supplementary.pdf]

Table S1. PRISMA checklist

TITLE			Location where item is reported
Title	1	Identify the report as a systematic review.	0
**ABSTRACT**			
Abstract	2	See the PRISMA 2020 for Abstracts checklist.	1
**INTRODUCTION**			
Rationale	3	Describe the rationale for the review in the context of existing knowledge.	2,3
Objectives	4	Provide an explicit statement of the objective(s) or question(s) the review addresses.	2,3
**METHODS**			
Eligibility criteria	5	Specify the inclusion and exclusion criteria for the review and how studies were grouped for the syntheses.	4
Information sources	6	Specify all databases, registers, websites, organisations, reference lists and other sources searched or consulted to identify studies. Specify the date when each source was last searched or consulted.	4
Search strategy	7	Present the full search strategies for all databases, registers and websites, including any filters and limits used.	4
Selection process	8	Specify the methods used to decide whether a study met the inclusion criteria of the review, including how many reviewers screened each record and each report retrieved, whether they worked independently, and if applicable, details of automation tools used in the process.	4
Data collection process	9	Specify the methods used to collect data from reports, including how many reviewers collected data from each report, whether they worked independently, any processes for obtaining or confirming data from study investigators, and if applicable, details of automation tools used in the process.	4
Data items	10a	List and define all outcomes for which data were sought. Specify whether all results that were compatible with each outcome domain in each study were sought (e.g. for all measures, time points, analyses), and if not, the methods used to decide which results to collect.	4
	10b	List and define all other variables for which data were sought (e.g. participant and intervention characteristics, funding sources). Describe any assumptions made about any missing or unclear information.	4
Study risk of bias assessment	11	Specify the methods used to assess risk of bias in the included studies, including details of the tool(s) used, how many reviewers assessed each study and whether they worked independently, and if applicable, details of automation tools used in the process.	4
Effect measures	12	Specify for each outcome the effect measure(s) (e.g. risk ratio, mean difference) used in the synthesis or presentation of results.	4
Synthesis methods	13a	Describe the processes used to decide which studies were eligible for each synthesis (e.g. tabulating the study intervention characteristics and comparing against the planned groups for each synthesis (item #5)).	4
	13b	Describe any methods required to prepare the data for presentation or synthesis, such as handling of missing summary statistics, or data conversions.	4
	13c	Describe any methods used to tabulate or visually display results of individual studies and syntheses.	4
	13d	Describe any methods used to synthesize results and provide a rationale for the choice(s). If meta-analysis was performed, describe the model(s), method(s) to identify the presence and extent of statistical heterogeneity, and software package(s) used.	4
	13e	Describe any methods used to explore possible causes of heterogeneity among study results (e.g. subgroup analysis, meta-regression).	4
	13f	Describe any sensitivity analyses conducted to assess robustness of the synthesized results.	4
Reporting bias assessment	14	Describe any methods used to assess risk of bias due to missing results in a synthesis (arising from reporting biases).	4

Certainty assessment	15	Describe any methods used to assess certainty (or confidence) in the body of evidence for an outcome.	4
**RESULTS**			
Study selection	16a	Describe the results of the search and selection process, from the number of records identified in the search to the number of studies included in the review, ideally using a flow diagram.	5,6
	16b	Cite studies that might appear to meet the inclusion criteria, but which were excluded, and explain why they were excluded.	5,6
Study characteristics	17	Cite each included study and present its characteristics.	5,6
Risk of bias in studies	18	Present assessments of risk of bias for each included study.	5,6 and supplementary tables 2 and 3
Results of individual studies	19	For all outcomes, present, for each study: (a) summary statistics for each group (where appropriate) and (b) an effect estimate and its precision (e.g. confidence/credible interval), ideally using structured tables or plots.	5,6
Results of syntheses	20a	For each synthesis, briefly summarise the characteristics and risk of bias among contributing studies.	5,6
	20b	Present results of all statistical syntheses conducted. If meta-analysis was done, present for each the summary estimate and its precision (e.g. confidence/credible interval) and measures of statistical heterogeneity. If comparing groups, describe the direction of the effect.	5,6
	20c	Present results of all investigations of possible causes of heterogeneity among study results.	5,6
	20d	Present results of all sensitivity analyses conducted to assess the robustness of the synthesized results.	5,6
Reporting biases	21	Present assessments of risk of bias due to missing results (arising from reporting biases) for each synthesis assessed.	5,6
Certainty of evidence	22	Present assessments of certainty (or confidence) in the body of evidence for each outcome assessed.	5,6
**DISCUSSION**			13
Discussion	23a	Provide a general interpretation of the results in the context of other evidence.	13
	23b	Discuss any limitations of the evidence included in the review.	13
	23c	Discuss any limitations of the review processes used.	13
	23d	Discuss implications of the results for practice, policy, and future research.	13
**OTHER INFORMATION**			
Registration and protocol	24a	Provide registration information for the review, including register name and registration number, or state that the review was not registered.	not reported
	24b	Indicate where the review protocol can be accessed, or state that a protocol was not prepared.	not reported
	24c	Describe and explain any amendments to information provided at registration or in the protocol.	not reported
Support	25	Describe sources of financial or non-financial support for the review, and the role of the funders or sponsors in the review.	14
Competing interests	26	Declare any competing interests of review authors.	14
Availability of data, code and other materials	27	Report which of the following are publicly available and where they can be found: template data collection forms; data extracted from included studies; data used for all analyses; analytic code; any other materials used in the review.	Not applicable

Table S2. Characteristics of included studies in systematic review and meta-analysis.

**CASE CONTROL STUDIES**								
**Author.<br>country.<br>Years**	**Inactive disease (ID)**<br>No. of subjects,<br>*median (IQR)**		**Active disease (AD)**<br>No. of subjects,<br>*median (IQR)**		**Healthy controls (HC)**<br>No. of subjects,<br>*median (IQR)**	**Laboratory Kit**	**p-value**	**Therapy**
			**Non Systemic (NS)**	**Systemic<br>(S)**				
Walscheid<br>Germany 2015<br>[5]	-		79<br>*(Not reported)*	-	24<br>*(Not reported)*	Not reported	AD vs HC p=0.009	Systemic immunosuppression 87.3%
Romano<br>Italy<br>2021<br>[6]	14<br>*24.8*<br>*(14.1-204.3)*		16<br>*29.6*<br>*(5.4-198.1)*	-	20	Quanta lite	AD vs ID p=0.29<br>p=0.06 AD+ID VS HC	Corticosteroids 6.6%<br>DMARDs 63.3%<br>Biological 6.6%
Boyoko<br>Ukraine<br>2017<br>[7]	87<br>*2.7*<br>*(1.7-4.0)*		122	17<br>*13.8*<br>*(5.8-26.0)*	10	Buhlmann	AD vs ID p=0.051	MTX 64%<br>Adalimumab 8.1%<br>Etanercept 4.3%<br>Prednisolone 8.7%<br>Tocilizumab 8.7%<br>Sulphasalazine 1.8%
Aljaberi<br>USA<br>2020<br>[8]	89<br>*14.71*<br>*(8.3-55.48)*		48<br>*(Not reported)*	22<br>*31.41*<br>*(37.08-47.17)*	Not enrolled	R&D System	ID vs NS p=0.9<br>ID vs S P<0.001	Data not reported
**LONGITUDINAL STUDIES**								
Chieti<br>Italy<br>2021	baseline	43<br>*6.89*<br>*(9.26)***	10<br>*9.47*<br>*(14.07)*	-		Calprest	AD vs ID p=0.92 at baseline	MTX 21%<br>Enbrel 24.5%<br>Adalizumab 32%<br>Remicade 3.7%
	6 months	35<br>*2.19*<br>*(1.135)***	10<br>*9.18*<br>*(22.85)*				AD vs ID P=0.07 at follow-up	
Barendreght<br>Netherlands<br>2020 [9]	Baseline<br>Cohort 1	54<br>1.183<br>(0.62-2.01)	32<br>5.39<br>(1.54-14.98)			Sanquin	Not reported	MTX monotherapy 59%<br>Etanercept monotherapy 2%

	6 months<br>Cohort I	3<br>0.39<br>(0.20-0.76)	19<br>0.36<br>(0.2-0.54)				P=0.68	Sulfalazine monotherapy 2%<br>MTX + anti TNF 11%
	Baseline<br>Cohort II	34<br>1.07<br>(0.62-2.4)	81<br>1.8<br>(1.16-2.32)				Not reported	MTX monotherapy 32%<br>Etanercept monotherapy 21%<br>Sulfalazine monotherapy 0%<br>MTX + anti TNF 13%
	6 months<br>Cohort II	28<br>1.51<br>(0.95-2.54)	53<br>2.02<br>(1.17-3.5)				P=0.15	
Hinze<br>Germany<br>2019 [10]	Baseline	106<br>*6.50*<br>*(0.49-38.9)*	24<br>*6.54*<br>*(2-27)*	-		Dianova	AD vs ID at baseline P=0.82	MTX 41.5%<br>Adalimumab 15.4%<br>Etanercept 80%<br>Infiximab 4.6%
	6 months	67<br>5.84<br>(0.71-5.0)	39<br>*5.44*<br>*(1.4-23.61)*	-	-		AD vs ID at follow-up P=0.36	
La<br>Belgium<br>2021 [11]	Baseline	45<br>*(Not reported)*	36<br>*(Not reported)*<br>-	-	11<br>*(Not reported)*	Buhlmann	AD vs ID<br>(* at baseline not reported differences among active disease) P<0.05<br>AD ca HC and ID P>0.001	Intraocular corticosteroids 49.4%, ongoing 9.9%<br>Oral corticosteroids 46.9%, ongoing 8.6%<br>MTX<br>88.9%, ongoing 75.3%<br>Others DMARDs 13.6%, ongoing 9.9%<br>Any Biologics 43.2%, ongoing 22.7<br>Anti-TFN 35.8%, ongoing 18.5%<br>Tocilizumab 9.9%, ongoing 3.7%<br>Anti IL 1 3.7%, ongoing 3.7<br>Abatecept 2.5, ongoing 1.2 %
	6 months	24<br>*12.13*<br>*(10.43)**	10<br>*27.44*<br>*(34.81)**	1	-		AD vs ID at follow-up P<0.05	
Boyko<br>Ukraine<br>2020 [12]	Baseline	49<br>*(Not reported)*	5<br>*(Not reported)*			Buhlmann	AD vs ID at baseline P=0.35	MTX 89%<br>Adalimumab + MTX 1.8 %<br>Etanercept +MTX 1.8 %<br>Delagil + MTX 1.8%<br>Tocilizumab +MTX 1.8%
	6 months	45<br>*1.5*<br>*(2-30)*	8<br>*1.70*<br>*(0.92-2.4)*				AD vs ID at follow-up P=0.619	

								Sulphasalazine 1.8%<br>Etanercept 1.8%
Anink<br>Netherlands<br>2015 [13]	Baseline	35<br>*1.17*<br>*(0.7-2.03)*	31<br>*2.28*<br>*(1.05-3.6)*	-		Buhlmann	AD vs ID at baseline P=0.005	Prendinsone 48%<br>MTX 97%<br>Other DMARD 30%<br>Etanercept 92%<br>Adalimumab 8%
	5 months	14<br>*(Not reported)*	12<br>*(Not reported)*				AD vs ID P=0.031	

(*) interquartile range
(**) Standard deviation
MTX metotrxate; DMARDs disease-modifying anti-rheumatic drugs

Table S3. Risk of bias of Case-control studies according to New-Castle Ottawa

	**Selection**				**Comparability**	**Exposure**			
Study	Is the case definition adequate?	Representativeness of the cases	Selection of Controls	Definition of Controls	Comparability of cases and controls on the basis of the design or analysis	Ascertainment of exposure	Same method of ascertainment for cases and controls	Non-Response rate	Total
Walscheid	*	*	-	*	*	*	*	*	7/8
Romano	*	*	-	*	*	*	*	*	7/8
Boyoko 2017	*	*	-	*	*	*	*	*	7/8
Aljaberi	*	*	-	*	*	*	*	*	7/8

Table S4. Risk of bias of longitudinal studies according to New-Castle Ottawa

	**Selection**				**Comparability**	**Exposure**			
Study	Representativeness of the exposed cohort?	Selection of the non exposed cohort	Ascertainment of exposure	Demonstration that outcome of interest was not present at start of study	Comparability of cohorts on the basis of the design or analysis	Assessment of outcome	Was follow-up long enough for outcomes to occur	Adequacy of follow up of cohorts	Total
Barendreght	*	*	*	-	*	*	*	*	7/8
La	*	*	*	-	*	*	*	*	7/8
Hinze	*	*	*	-	*	*	*	*	7/8
Boyoko 2020	*	*	*	-	*	*	*	*	7/8
Annik	*	*	*	-	*	*	*	*	7/8

	ES	95% CI	W	Sig.	N
Chieti 2021	0.01	-0.54 / 0.55	64.03%	0.980	54
Romano 2021	0.39	-0.33 / 1.12	35.97%	0.285	30
Overall (random-effects model)	0.15	-0.29 / 0.58	100.00%	0.509	84

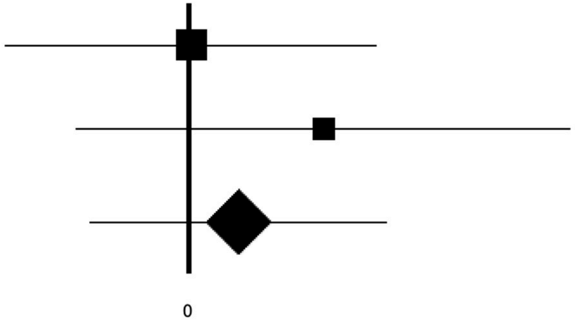

(**a**)

	ES	95% CI	W	Sig.	N
Chieti 2021	0.42	-0.13 / 0.96	63.10%	0.137	54
Romano 2021	0.00	-0.72 / 0.72	36.90%	1.000	30
Overall (random-effects model)	0.26	-0.17 / 0.70	100.00%	0.237	84

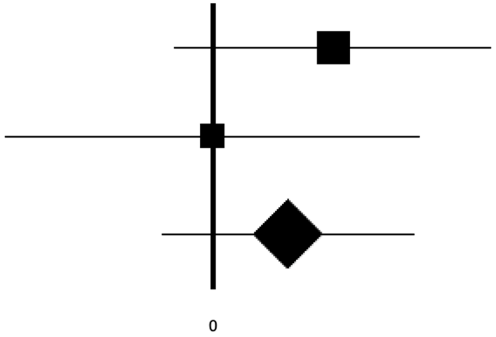

(**b**)

Figure S1. Calprotectin levels according to clinical criteria (**a**) and echography score (**b**).
